# Tobacco smoke induces changes in IL-1 family in bronchial epithelial cells obtained from asthmatic individuals

**DOI:** 10.1186/1710-1492-10-S2-A55

**Published:** 2014-12-18

**Authors:** Valérie Gagné-Ouellet, Éric Jacques, Anne-Marie Boucher-Lafleur, Sophie Plante, Luigi Bouchard, Jamila Chakir, Catherine Laprise

**Affiliations:** 1 Département des sciences fondamentales, Université du Québec à Chicoutimi, Chicoutimi, Quebec, G7H 2B1, Canada; 2 Centre de recherche, Institut Universitaire de Cardiologie et de Pneumologie, Ste-Foy, Quebec, G1V 4G5, Canada; 3 Département de biochimie, Université de Sherbrooke, Sherbrooke, Quebec, J1K 2R1, Canada; 4 ECOGENE-21 et la Clinique Lipidique, Hôpital de Chicoutimi, Chicoutimi, Quebec, G7H 5H6, Canada

## Background

Exposure to tobacco smoke (ETS) induces epigenetic modifications including DNA methylation [1]. In asthma, it has been shown that those modifications affect immune cell differentiation by downregulating expression of specific pro-inflammatory cytokines [2-4]. Interleukin 1 (IL-1) is recognized to be increased in asthma [5] and by cigarette smoke [5,6]. Based on previous genetic association [7,8] and DNA methylation signature of receptors in asthma and/or atopy the aim of this study is to evaluate the changes in expression and methylation pattern induced by ETS for IL-1 subunit alpha (*IL-1A*) and beta (*IL-1B*), receptors type I (*IL-1R1*), type II (*IL-1R2*) and antagonist (*IL-1RA*) and for interleukin 33 (*IL-33*) in lung tissue.

## Methods

Primary epithelium cells isolated from bronchial biopsies of mild asthmatics and non-asthmatics individuals were exposed to whole tobacco smoke according to method described [9]. Level of mRNA was measured by qRT-PCR and methylation was assessed by bis-pyrosequencing for *IL-1A*, *IL-1B*, *IL-1R1*, *IL-1R2*, *IL-1RA* and *IL-33*.

## Results

ETS increased mRNA level of *IL-1A and IL-1B* in both asthmatic and non-asthmatic individuals. *IL-33* showed a significant decrease in gene expression following ETS in asthmatic individuals but not in non-asthmatics*. IL-1R1* was decreased in non-asthmatic individuals but no change was observed in asthmatics. *IL-1R2* and *IL-1RA* increased in both asthmatic and non-asthmatic individuals. We observed DNA methylation differences in *IL-1R1* promoter between ETS and non-ETS cells.

## Conclusions

Modifications of genes expression induced by tobacco smoke could modify IL-1 family resulting in an increase of inflammation in lung tissues of asthmatic and non-asthmatic individuals. These changes may be induced by DNA methylation. Efforts to better interpret and integrate data from genetics and epigenetics are needed to better understand the biology of asthma as well as a better comprehension of the impact of tobacco smoke in the inflammatory component of asthma.
